# Undergraduate palliative care teaching in Swiss medical faculties: a nationwide survey and improved learning objectives

**DOI:** 10.1186/s12909-015-0485-0

**Published:** 2015-11-27

**Authors:** S. Eychmüller, M. Forster, H. Gudat, U. M. Lütolf, G. D. Borasio

**Affiliations:** 1 Center for Palliative Care, University hospital, Inselspital Berne, Freiburgstrasse 28, CH- 3010 Berne, Switzerland; 2 Center for Palliative Care, Cantonal Hospital St. Gallen, St. Gallen, Switzerland; 3 Hospice “Im Park”, Arlesheim, Switzerland; 4 Department of Radio-Oncology, University Hospital Zurich (until Dec 2012), Zurich, Switzerland; 5 Service de Soins Palliatifs, University Hospital Lausanne, Lausanne, Switzerland

## Abstract

**Background:**

In 2007, a first survey on undergraduate palliative care teaching in Switzerland has revealed major heterogeneity of palliative care content, allocation of hours and distribution throughout the 6 year curriculum in Swiss medical faculties. This second survey in 2012/13 has been initiated as part of the current Swiss national strategy in palliative care (2010 – 2015) to serve as a longitudinal monitoring instrument and as a basis for redefinition of palliative care learning objectives and curriculum planning in our country.

**Methods:**

As in 2007, a questionnaire was sent to the deans of all five medical faculties in Switzerland in 2012. It consisted of eight sections: basic background information, current content and hours in dedicated palliative care blocks, current palliative care content in other courses, topics related to palliative care presented in other courses, recent attempts at improving palliative care content, palliative care content in examinations, challenges, and overall summary. Content analysis was performed and the results matched with recommendations from the EAPC for undergraduate training in palliative medicine as well as with recommendations from overseas countries.

**Results:**

There is a considerable increase in palliative care content, academic teaching staff and hours in all medical faculties compared to 2007. No Swiss medical faculty reaches the range of 40 h dedicated specifically to palliative care as recommended by the EAPC. Topics, teaching methods, distribution throughout different years and compulsory attendance still differ widely. Based on these results, the official Swiss Catalogue of Learning Objectives (SCLO) was complemented with 12 new learning objectives for palliative and end of life care (2013), and a national basic script for palliative care was published (2015).

**Conclusion:**

Performing periodic surveys of palliative care teaching at national medical faculties has proven to be a useful tool to adapt the national teaching framework and to improve the recognition of palliative medicine as an integral part of medical training.

## Background

Based on recommendations published by the European Association for Palliative Care (EAPC) [1], palliative care content has been integrated into the medical curricula of several European countries in recent years. A national survey on palliative care education in Swiss undergraduate medical curricula which took place in 2007 was published under the title: “A case of too little, too early” [2]. The result of this survey showed a very heterogeneous picture of palliative care undergraduate training. Although most curricula covered some domains of palliative care (e.g. ethics in end of life care), total teaching hours, allocation within the 6-year study plan and integration of palliative care specialists as teachers varied dramatically within the five Swiss medical faculties.

Since 2010, the Swiss Federal Office for public health has implemented a National Strategy for Palliative Care [3]. Within this strategy, the Health Office mandated a group with experts from each medical faculty in Switzerland to assess the status of undergraduate training in palliative care at Swiss Medical faculties. These data should serve as a basis for the elaboration of a proposal on how to improve palliative care training in Switzerland. Most importantly, the proposal should include integration of palliative care topics into the Swiss Catalogue of Learning Objectives (SCLO) for undergraduate medical training similar to German recommendations [4, 5].

## Methods

### Survey

In order to allow comparison to the 2007 survey, we chose an analogous approach. In June 2012, a questionnaire with closed and open items was sent electronically to two key persons within each of the five Swiss medical faculties (Universities of Basel, Bern, Geneva, Lausanne and Zurich). One was addressed to the faculty member responsible for palliative care training, the other to the vice-dean for medical education and asking for one single feedback per faculty. The questionnaire consisted of eight sections as in the previous survey: basic background information, current content and hours in dedicated palliative care blocks, current palliative care content in other courses, topics related to palliative care presented in other courses, recent attempts at improving palliative care content, palliative care content in examinations, challenges, and overall summary.

The results of the survey were mapped against a) the results of our national survey from 2007, and b) selected items from the EAPC recommendations [1] regarding content, teachers, methods and dedicated palliative care blocks.

### Proposal development

The same working group was asked in 2013 to develop, based on the results of the survey, learning objectives pertaining to palliative care for inclusion in the SCLO. The group chose to use an iterative process involving experts from all Swiss Medical Faculties and large clinical centers with palliative care expertise. The process involved three rounds of sampling and prioritizing the teaching topics structured by subheadings related from the EAPC syllabus (round 1 group work sampling, round 2 individual, independent ranking, round 3 final group discussion and agreement). The topics were put in relation with existing general learning objectives in the SCLO and international recommendations. A national basic script for palliative care based on the new learning objectives was finally developed in 2014 [6].

Ethics: since the survey did not include patient data or patient intervention, ethical approval has been deemed as “not applicable” (Cantonal ethical committee Bern, Switzerland, 2012), which means that consent by participants was deemed unnecessary.

## Results

Apart from one faculty with the vice-dean responding (responsible for palliative care teaching at that time), the survey was answered by dedicated palliative care specialists as responsible or co-responsible persons for palliative care teaching at the faculty. All respondents hold an academic position at their university.

All five medical faculties base their teaching on an organ-system based curriculum, with differences of the integration of problem-based teaching units. There have not been important changes over the last 5 years in terms of regulations and academic recognition of palliative medicine: despite an increased number of defined academic positions, the number of academic chairs (*n* = 1) has remained unchanged. Specific learning objectives for palliative care cannot be found within the SCLO, nor have learning outcomes been harmonized among the medical faculties.

### Palliative care content in dedicated palliative care blocks

**Fig. 1:**
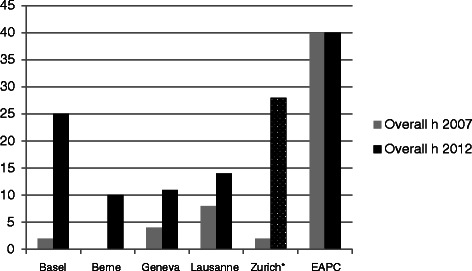
Mandatory hours in dedicated palliative care blocks 2007 and 2012. Legend: *Zurich: mandatory for 20 % of students in 3^rd^ or 4^th^ academic year; ** Practical year

Most content recommended by the EAPC is covered within the current curricula, but the numbers of hours differ widely (Fig. 1). In addition, allocation of palliative care content throughout the six years of medical training differs widely (Table 1).

**Table 1:** allocation of palliative care blocks throughout all academic years

	Basel	Berne	Geneva	Lausanne	Zurich^a^	EAPC^c^
	N	N	N	N	N	N
1st academic year	2	0	0	0	0	
2nd academic year	0	0	5	0	0	
3rd academic year	21	0	0	0	28	
4th academic year	0	0	6	14	0	
5th academic year	0^b^	10	0	0	0	
6th academic year	2	0^b^	0^b^	0^b^	0^b^	
Overall h 2012	25	10	9	14	28	40
Overall h 2007	2	0	4	8	2	40

^a^*Zurich:* mandatory *for 20 % of students* in 3^rd^ or 4^th^ academic year; ^b^Practical year; ^c^European Association for Palliative Care

### Mandatory palliative care blocks

The number of mandatory hours for medical students is highest at the Universities of Basel (25 h). and lowest in Berne (10 h) and Geneva (9 h). In Zurich, 28 h are mandatory for only 20 % of students (by choice). The number of mandatory hours spent in courses with so-called “embedded palliative care content” or in “other courses” is difficult to determine, because palliative care content cannot always be clearly identified.

Compared to 2007, all faculties show an increase of the total number of mandatory hours for palliative care training. However, in Lausanne mandatory theoretical teaching hours were reduced significantly in 2010/11, despite excellent evaluation by the students, in the context of a restructuration of the undergraduate curriculum and a vacancy of the chair in palliative medicine.

### Teachers and instructional methods

In all medical faculties, staff with specialized or advanced competency in palliative care is responsible for offering lectures and courses, which was not the case five years earlier. However, only one faculty integrates other professions like social workers or physiotherapists into the curriculum. Small group learning and clinical rotation/bedside teaching, which may be essential for self-reflected learning in this challenging domain of medicine, cannot be offered on a regular basis in most universities mainly due to shortage of trained palliative care specialists.

### Topics/ Syllabus

**Fig. 2:**
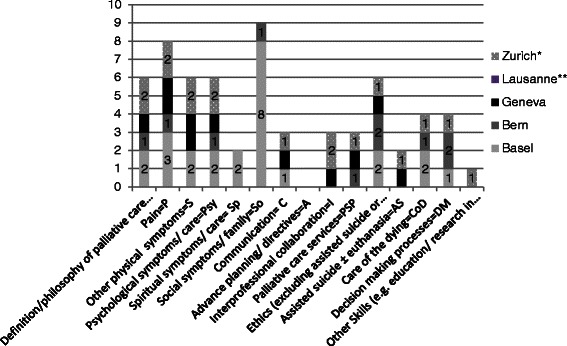
content in numbers of hours covered in dedicated palliative care blocks (all medical faculties). Legend: *Mandatory for 20 % of students (+9 h in clinical rotation); **various topics in clinical rotation

Palliative care content in dedicated palliative care blocks is shown in Fig. 2. Social symptoms and family issues seem to be the most frequent topics associated with palliative care content, followed by physical symptoms including pain, and ethics. This situation does not reflect the EAPC recommendations, which are shown in Table 2. Comparison to 2007 is difficult because content analysis in 2007 has been made for dedicated palliative care blocks and topics covered in other blocks in combination.

**Table 2:** EAPC recommendation 2013: suggested percentages for the palliative care topics to be covered in medical undergraduate curricula (1)

Basics of Palliative Care	5 %
Pain and symptom management	50 %
Psychosocial and spiritual aspects	20 %
Ethical and legal issues	5 %
Communication	15 %
Teamwork and self-reflection	5 %

### Clinical rotations

Only Lausanne offers a mandatory 2-day clinical rotation in palliative care (inpatient units and/or home care teams, both hospital-based and community-based). All other faculties encourage students to participate in clinical rounds in various specialized palliative care settings (hospice, palliative care unit), but only for a limited number of students (between 2 and 50).

### Courses on topics relevant to palliative care but not designated as such

At the University of Basel intensive training is offered in communication skills including video-feedback and OSCE. Within these courses, difficult situations concerning end of life communication are integrated. In other medical faculties such as Geneva and Zurich, various topics with link to palliative care are integrated into general practice training, e.g. collaboration with district nurses, prescriptions of opioids and ethical and decision-making issues.

### Palliative care content in examinations

Compared to 2007, Berne and Zurich included multiple-choice type questions in the 5^th^ year examination or in the final examination (faculty examination) respectively.

Geneva has newly introduced multiple-choice type questions in the 4^th^ year examination and Basel requires multiple-choice type questions for symptom control, care of the dying, decision-making, and ethics in the 6^th^ year. On a national level, palliative care multiple-choice type questions have been included into the national board examination at the end of year 6.

### Recent attempts to improve palliative care education and challenges

Except for Zurich where only the current revision of the 6^th^ year curriculum will integrate palliative care issues, academic leaders for palliative care within the medical faculties have tried to increase the number of hours/content related to their topic during the last 5 years. In Berne, a dedicated palliative care block has been restructured and is now offering multi-professional teaching and systematic coverage of palliative care learning objectives following EAPC content splitting in the 5^th^ year. Additional mandatory seminars in conjunction with ethics, geriatrics and neonatology have been put in place in 2013. In Geneva, attempts have been made to increase the responsibility and number of tutors for mandatory palliative care courses. In Basel, multi-professional seminars have been proposed, but not yet implemented. Lausanne has succeeded in 2014 to resume earlier “lost hours” for dedicated palliative care seminars, which are now offered with multiprofessional teaching.

A major challenge and strategic priority for the future, as reported by palliative care faculty members from all medical faculties, is to create sufficient academic staff to provide high quality education in this new domain. Faculties struggle with getting more tutors for small group seminars and a sufficient number of academic teachers with specialized palliative care background. The permanent double challenge of defending existing and finding new training hours in the curriculum is a main concern in all medical faculties. This reflects the concern to get adequate recognition for palliative care as an academic discipline. Additional challenges include the integration of nurses and other health care professionals as teachers, as well as finding a sufficient number of specialized institutions such as hospices and mobile teams to perform clinical rotations. The number of hours with dedicated palliative care blocks is far too low compared to EAPC recommendations, as is the representation of palliative care content in examinations.

### Proposal development

The iterative process yielded 58 learning objectives, which were then prioritized by the participants and put in relation with existing general learning objectives in the SCLO. This was necessary in order to ensure internal coherence with the existing learning objectives. Agreement among the experts from the five medical faculties was highest in general objectives/ basics, pain management, psychosocial support, and self-reflection/ self-care. The resulting proposal is shown in Table 3. The Swiss Medical Interfacultary Conference has accepted the proposal in November 2012.

**Table 3:** Proposal for new palliative care learning objectives: The new objectives (right column) are related to general learning objectives already present in the Swiss Catalogue of Learning Objectives SCLO (middle column)

Topics	Basis for the proposal in the SCLO	New palliative care learning objectives
Pain- and symptom- manage-ment	G ME^a^ 41: The physician explains and applies the principles of therapeutics in treatment of pain, palliative and end-of-life care	The physician understands the concept of total pain and its impact on palliative care planning.
		The physician understands the pathophysiology of the main symptoms in severe disease (e.g. dyspnea, pain nausea/vomiting, delirium, anxiety) and applies this knowledge in his choice of treatment.
		The physician applies specific symptom assessment systems to assess and monitor frequent symptoms in palliative care.
		The physician understands the principles of adequate prescription of the non-pharmacological and pharmacological treatments (including e.g. opioids, sedatives, and neuroleptics) required for symptom control in the palliative phase.
Dying and death	G ME 41: The physician explains and applies the principles of therapeutics in treatment of pain, palliative and end-of-life care.	The physician is able to explain the normal physiology of the dying process to the patients and the family members.
	G CM^a^ 3: The physician chooses a suitable setting with necessary support when giving complicated or bad news	
Change in treatment goals at the end of life	G CM 9: The physician clarifies the patient’s expectations and requests for the encounter and elicits information on both the somatic and psychological aspects of her/his symptoms and complaints as well as the patient’s situation, her/his understanding and concerns, social and cultural background and illness experience	The physician understands the importance of advance care planning. He assists discussion on decision-making at the end of life and supports the definition of patients’ preferences and acceptable outcomes.
		The physician understands the legal basis and the relevance of advance directives, as well as the role of the health care proxy.
	G ME 33: The physician explains criteria for issuing ‘Do Not Attempt Resuscitation’-orders and the level of experience required to issue them.	
	G PR^a^ 23: The physician identifies the ethical principles in decisions regarding discontinuation or withholding of life-support measures.	
Physician’s own limits	G PR 9: The physician demonstrates an appropriate, caring attitude with consistently high standards of professional behavior, including honesty, integrity, accountability, commitment, compassion, empathy and altruism.	The physician is aware of his own limits and his own mortality.
	G PR 10: The physician maintains an appropriate balance between personal and professional roles and shows awareness of possible conflicts of interest.	
Multiprofes-sionality and home care	G ME 1: The physician demonstrates clear history taking and communication with patients, their families and other carers and seeks information from other sources, differentiating the concepts of ‘illness’ as the patient’s story and of ‘disease’ as the medical history of a health disorder	The physician is able to run a family meeting. He/she knows how to integrate other professionals when needed to address the physical, psychosocial and spiritual needs of severely ill patients and their significant others.
		The physician shows an awareness of transcultural issues at the end of life.
	G ME 8: The physician takes into consideration relevant context and background of the patient, including family, social, cultural and spiritual factors.	The physician shows a positive attitude towards multiprofessional home care in the last phase of life and the importance of adapting the care to suit the environment and the patient’s needs and wishes.
	G ME 27: The physician demonstrates an understanding of the social and cultural background of patients and takes it into account in her/his clinical work.	
	G ME 23: The physician pays attention to the importance of continuity of care and of patient information transfer e.g. from inpatient to outpatient setting.	

^a^GME: General objective as medical expert

GCM: General objective as communicator

GPR: General objective as professional

## Discussion

Compared to 2007, training in palliative medicine in Swiss medical faculties has shown some improvements, but there are still major inconsistencies and heterogeneity. Total hours of palliative care content have increased, but are still far from the 40 h recommended by the EAPC. This number of hours can only be reached in some medical faculties if the hours of dedicated palliative care blocks and palliative care content in other courses are taken together. Heterogeneity in palliative care teaching can be explained by the lack of common understanding and definition of learning objectives, as was the case in the US until recently [7, 8].

Worldwide, training in palliative care and end of life care has gained growing recognition as being a mandatory and important part of medical curricula, partly due to the increased recognition of current and future sociodemographic changes [9]. Due to its small size and low number of medical faculties, Switzerland may be regarded as an educational laboratory regarding implementation of end of life issues in medical training as it has been reported from other countries [10]. However, our approach may not be directly transferable to other countries, due to local specificities concerning e.g. a high autonomy of individual faculties in the creation of their curricula.

One reason for the improved situation in 2012/13 compared to five years earlier lies in the National Strategy for Palliative Care 2010–2012 [3], which has been recently prolonged until end 2015 because of the need for a more sustainable implementation of palliative care into regular health care. A similar development has taken place in Canada with the Canadian strategy on palliative and end of life care in the late nineties and early years of the 21st century, which resulted in the nationwide implementation of a defined palliative care curriculum for medical students initially edited in 1993 [11]. In Canada as well as in Australia [12], the introduction of palliative care as a specialty or subspecialty – not only in medicine but also in other disciplines – has led to an increased availability of highly qualified academic teachers. Unfortunately, the number of academic chairs has remained unchanged in Switzerland - just one single chair to date. Thanks to the momentum provided by the national strategy for palliative care, we have good reasons to believe that specialist training for palliative medicine will be available in Switzerland before 2016.

## Conclusions

The aim of the study was to provide facts and figures needed to develop a core set of learning objectives specific for palliative care. The proposal made by the study group achieved broad acceptance by the Swiss Medical Interfaculty Conference. National adaptation of internationally available guidance [13] is needed to reach such a goal, as has been shown by a similar iterative process proposed by the Competencies Work Group of the American Board of Hospice and Palliative Medicine [14]. Thus, performing periodic surveys of palliative care teaching at national medical faculties has proven to be a useful tool to adapt the national teaching framework and to improve the recognition of palliative medicine as an integral part of medical training. Students ask for professionalism when dealing with difficult situations such as decision making in serious illness or caring for dying patients. Formal training and exposure to patients leave students dramatically better prepared for these frequent clinical situations [15]. An inter-faculty students’ assessment of palliative care competencies is needed to complete the picture in the future.

The agreement on new learning objectives in palliative and end of life care for all Swiss medical schools based on the results of this second national survey has prompted various projects and developments. It has already led to the allocation of eight new mandatory teaching hours in palliative care at the University of Lausanne starting 2014. A national basic script on palliative care for medical students has been published in 2015 with the support of the national strategy.

This project has brought together politicians, deans, palliative experts, other professionals and medical students. Its results are in our opinion a valuable step towards future health care professionals who will be better prepared for the challenges of an aging population.
